# Trends in the Prevalence of Cardiometabolic Multimorbidity in the United States, 1999–2018

**DOI:** 10.3390/ijerph19084726

**Published:** 2022-04-14

**Authors:** Xunjie Cheng, Tianqi Ma, Feiyun Ouyang, Guogang Zhang, Yongping Bai

**Affiliations:** 1 Department of Geriatric Medicine, Center of Coronary Circulation, Xiangya Hospital, Central South University, Changsha 410008, China; matianq@csu.edu.cn; 2 National Clinical Research Center for Geriatric Disorders, Xiangya Hospital, Central South University, Changsha 410008, China; 3 Department of Social Medicine and Health Management, Central South University, Changsha 410078, China; feiyun0716@csu.edu.cn; 4 Department of Cardiovascular Medicine, Xiangya Hospital, Central South University, Changsha 410008, China; zhangguogang@csu.edu.cn; 5 Department of Cardiovascular Medicine, The Third Xiangya Hospital, Central South University, Changsha 410013, China

**Keywords:** cardiometabolic disease, multimorbidity, epidemiology, NHANES

## Abstract

Cardiometabolic multimorbidity (co-existence of ≥1 cardiometabolic diseases) is increasingly common, while its prevalence in the U.S. is unknown. We utilized data from 10 National Health and Nutrition Examination Survey (NHANES) two-year cycles in U.S. adults from 1999 to 2018. We reported the age-standardized prevalence of cardiometabolic multimorbidity in 2017–2018 and analyzed their trends during 1999–2018 with joinpoint regression models. Stratified analyses were performed according to gender, age, and race/ethnicity. In 2017–2018, the prevalence of cardiometabolic multimorbidity was 14.4% in the U.S., and it was higher among male, older, and non-Hispanic Black people. The three most common patterns were hypertension and diabetes (7.5%); hypertension, diabetes, and CHD (2.2%); and hypertension and CHD (1.8%). During 1999–2018, the prevalence of cardiometabolic multimorbidity in U.S. adults increased significantly, with an averaged two-year cycle percentage change (AAPC) of 3.6 (95% CI: 2.1 to 5.3). The increasing trend was significant for both genders, most age groups except for 60–79 years, and non-Hispanic White people. For common patterns, the trend was increasing for hypertension and diabetes and hypertension, diabetes, and CHD, while it was decreasing for hypertension and CHD. Our findings provide evidence that cardiometabolic multimorbidity has risen as an austere issue of public health in the U.S.

## 1. Introduction

Cardiometabolic multimorbidity is a comorbidity pattern, which means the co-occurrence of ≥2 cardiometabolic diseases in an individual has risen as a global issue for public health [1]. Evidence suggested that cardiometabolic multimorbidity was associated with a higher risk of cognitive dysfunction [2], depression [3], infection and poor prognosis of COVID-19 [4,5], and all-cause mortality [6]. At the age of 60, a history of concomitant diabetes, myocardial infarction, and stroke was associated with a reduced life expectancy by 15 years, compared with the disease-free condition [6].

Along with the global tendency of population aging, it is not beyond expectation that cardiometabolic multimorbidity is increasingly common since most cardiometabolic diseases, such as coronary heart disease (CHD), stroke, diabetes, and hypertension, have overlapping risk factors, etiology, and bidirectional interactions [1,7]. For instance, diabetes or hyperglycemia is a major risk factor for atherosclerotic diseases, including CHD, cerebrovascular disease, and peripheral arterial disease [8], and it also accelerates the development of restenosis after endovascular therapeutic procedures of atherosclerotic diseases [9], which is attributed to hyperglycemia-related excessive oxidative stress, inflammation, and endothelial dysfunction [8]. Similarly, hypertension is also an important risk factor for cardiovascular diseases and is bidirectionally associated with diabetes [10,11]. Epidemiologically, it has been reported that cardiometabolic multimorbidity affected nearly 25% of patients with cardiometabolic disease in Canada [12] and South Asia [13], and its prevalence doubled in the general Chinese population over 5 years after 2010 [14]. However, less is known about the prevalence and trend of cardiometabolic multimorbidity in the United States.

To address this issue, we utilized data from the National Health and Nutrition Examination Survey (NHANES) 1999–2018 cycles to examine the latest prevalence (2017–2018) of cardiometabolic multimorbidity (overall and stratification according to disease number and specific patterns) and its trend during the past two decades.

## 2. Materials and Methods

### 2.1. Study Sample

The data source was the cross-sectional NHANES of 10 two-year cycles from 1999 to 2018. The NHANES recruited a representative sample of the non-institutionalized civilian population of the U.S. using a complex, stratified, multistage probability-cluster sampling design, and it was approved by the National Center for Health Statistics Research Ethics Review Board. The survey data are publicly available on the Internet and CD-ROMs for researchers and data users globally [15]. Providing written informed consent, participants finished a questionnaire at home and completed an interview and physical examination at a mobile examination center (MEC). Biological specimens were also collected for laboratory analyses. Detailed design and operations of NHANES were published previously [16]. For this study, we enrolled participants aged 20 years and older with available information from interviews, physical examination, and fasting laboratory data (*n* = 22,266).

### 2.2. Measures

Information on age, gender, race/ethnicity, and medical conditions was derived from questionnaires and interviews. Age was categorized as 20–39, 40–59, 60–79, and ≥80 years old. Race/ethnicity was self-reported in the standardized questionnaires and was classified as non-Hispanic white, non-Hispanic black, Hispanic, and other race/ethnicity. Blood pressure (BP, mmHg) was measured in MECs with standard protocols, and averaged systolic and diastolic BP were calculated according to the analytic notes of the NHANES [17]. Glycated hemoglobin (HbA1c, %) was measured and standardized using the Diabetes Control and Complications Trial method. In MECs, a random subset of participants was examined in the morning session, and the fasting plasma glucose levels of people who fasted for 8–24 h were measured. Glucose data from the 2005–2006, 2007–2008, 2015–2016, and 2017–2018 cycles were calibrated following the recommended method of the National Center for Health Statistics because of the alterations in the laboratory method, equipment, or site [18].

So far, there has been no consensus about the definition of cardiometabolic multimorbidity. In published studies, cardiometabolic diseases, including CHD, diabetes, stroke, hypertension, dyslipidemia, and chronic kidney disease, were used to ascertain the comorbidity status [4,6,7,12]. In consideration of both sample sizes of certain diseases in the NHANES and clinical practice, four common cardiometabolic diseases, including CHD, diabetes, stroke, and hypertension, were involved to assess the status of cardiometabolic multimorbidity. Hypertension was defined as a self-reported history of hypertension, a mean systolic BP ≥ 130 mmHg, a mean diastolic BP ≥ 80 mmHg, or taking a prescription for hypertension. Diabetes was defined as a self-reported history of diabetes, a fasting plasma glucose level ≥ 126 mg/dL, or HbA1c level ≥ 6.5%. CHD was defined as a self-reported history of CHD, angina, or heart attack. Stroke was defined as a self-reported history of stroke. Participants who had ≥two conditions of the four diseases above were thought to have cardiometabolic multimorbidity, with disease numbers recorded. According to specific combinations of concurrent diseases, the comorbidity was also classified exclusively into 11 patterns: (1) hypertension and diabetes; (2) hypertension, diabetes, and CHD; (3) hypertension and CHD; (4) hypertension and stroke; (5) hypertension, stroke, and diabetes; (6) hypertension, stroke, CHD, and diabetes; (7) hypertension, stroke, and CHD; (8) CHD and diabetes; (9) stroke and diabetes; (10) stroke and CHD; and (11) stroke, CHD, and diabetes.

### 2.3. Statistical Analyses

All statistical analyses were performed following the analytic guidelines provided by the National Center for Health Statistics [18,19], accounting for the complex survey design, strata, and sample weight of the NHANES, which generated estimates for the total non-institutionalized civilian population of the U.S. First, the age-standardized prevalence (per two-year cycle) of cardiometabolic multimorbidity (overall, two concurrent, and ≥three cardiometabolic diseases) were estimated in the entire U.S. adult population separately and then stratified by age, gender, and race/ethnicity. Corresponding sampling weights for the fasting laboratory tests were used for each cycle to account for non-response, non-coverage, and unequal probabilities of selection to generate representative estimates of the total of non-institutionalized U.S. adults. This study used the 2000 census as the standard population. The prevalence of common patterns of cardiometabolic multimorbidity (case counts ≥ 20 per cycle) in the entire population was reported. The latest prevalence of this comorbidity and common patterns in the U.S. adults were presented. In addition, the affected population counts (in millions) were calculated using the population totals during 2017–2018 [20]. Second, joinpoint regression models were applied to assess the trends in the prevalence of cardiometabolic multimorbidity in the total population and among subgroups from 1999 to 2018 [19]. With the age-standardized prevalence and standard error from 10 NHANES two-year cycles entered, Joinpoint software assessed whether there were statistically significant alterations (i.e., joinpoints) in the trends. The program started with 0 joinpoints and tested whether ≥1 joinpoints (in this study, maximum 1) were significant to be added to the model. Two-year cycle percentage change (APC) and averaged APC (AAPC), corresponding 95% CI, and *p* for the trend were generated to assess the overall trends in the prevalence of cardiometabolic multimorbidity during the past two decades. In the current analyses, the results were tagged with “unreliable” when the corresponding case counts were <20 since the results estimated based on such a small sample size were prone to be influenced by chance [21]. The data were analyzed with R version 4.0.4, STATA version 17, and Joinpoint Regression Program version 4.9.0.0 (National Cancer Institute). A two-sided *p* < 0.05 was used to determine statistical significance.

## 3. Results

### 3.1. Prevalence of Cardiometabolic Multimorbidity in the U.S., 2017–2018

A total of 2291 participants aged 20–85 years from the NHANES 2017–2018 cycle were enrolled in our analysis of the latest prevalence of cardiometabolic multimorbidity. The weighted mean age was 48.23 years, 48.3% of the participants were male, and 61.9% were non-Hispanic white.

The latest prevalence of cardiometabolic multimorbidity was 14.4% in the U.S., which indicated that 34.27 million adults were affected during 2017–2018 (Table 1). The prevalence was 15.6% (37.23 million) among men and 13.4% (31.91 million) among women and increased with age. People aged 20–39 years had the lowest prevalence of 2.3%, which increased to the highest prevalence of 42.9% among people aged ≥ 80 years. In terms of race/ethnicity, non-Hispanic Black adults had the highest prevalence of cardiometabolic multimorbidity. When analyzed according to disease numbers, 10.7% (25.47 million) of U.S. adults had two cardiometabolic diseases, 3.7% (8.8 million) had ≥three diseases, and the subgroups with the highest prevalence of both conditions were consistent: male, aged ≥ 80 years, and non-Hispanic Black. The three most common patterns were hypertension and diabetes (7.5%, 18.02 million); hypertension, diabetes, and CHD (2.2%, 5.35 million); and hypertension and CHD (1.8%, 4.32 million).

### 3.2. Trend in the Prevalence of Cardiometabolic Multimorbidity in the U.S., 1999–2018

**Figure 1:**
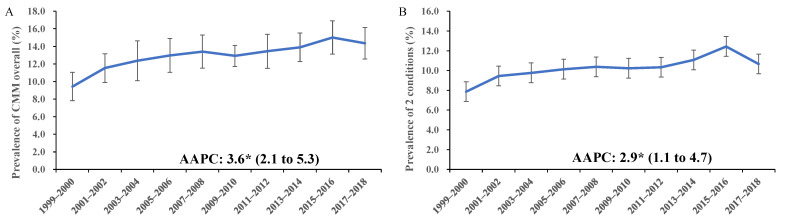

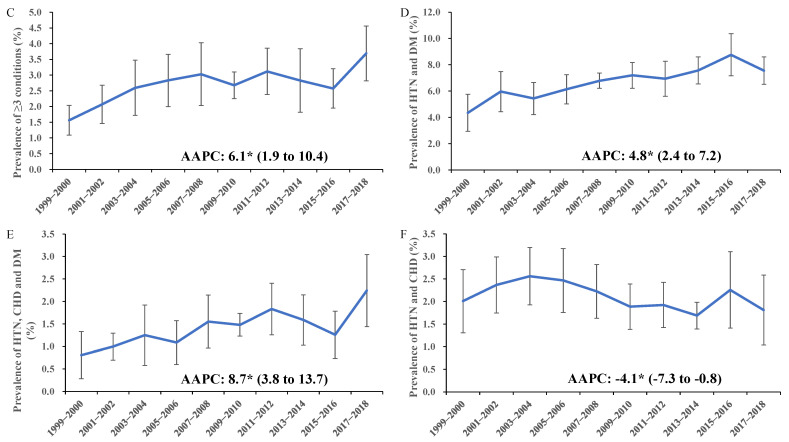
Trends in the age-adjusted prevalence of cardiometabolic multimorbidity and specific patterns among U.S. adults, 1999–2018. Notes: The AAPC was derived from joinpoint regression models to assess the averaged trends in the prevalence of comorbidity. Panel (**A**): For CMM overall; Panel (**B**): For two concurrent cardiometabolic diseases; Panel (**C**): For ≥three concurrent cardiometabolic diseases; Panel (**D**): For CMM pattern, HTN, and DM; Panel (**E**): For CMM pattern, HTN, CHD, and DM; Panel (**F**): For CMM pattern, HTN, and CHD. CMM, cardiometabolic multimorbidity; HTN, hypertension; DM, diabetes mellitus; CHD, coronary heart disease; AAPC, averaged two-year cycle percentage change. * *p* for trend <0.05.

From 1999 to 2018, the overall prevalence of cardiometabolic multimorbidity in U.S. adults increased from 9.4% to 14.4%, with an estimated averaged two-year cycle percentage change (AAPC) of 3.6 (95% CI: 2.1 to 5.3, *p* = 0.001) (Figure 1A). The prevalence of two concurrent and ≥three conditions increased from 7.9% to 10.7% (AAPC: 2.9, 95% CI: 1.1 to 4.7) and from 1.6% to 3.7% (AAPC: 6.1, 95% CI: 1.9 to 10.4), respectively (Figure 1B,C). For the three common patterns of cardiometabolic multimorbidity, the trends in their prevalence were distinct (Figure 1D–F). Generally, the prevalence of hypertension and diabetes and hypertension, diabetes, and CHD increased steadily, with AAPCs of 4.8 (95% CI: 2.4 to 7.2) and 8.7 (95% CI: 3.8 to 13.7), while the trend of hypertension and CHD was decreasing (AAPC: −4.1, 95% CI: −7.3 to −0.8). The detailed values and 95% CI of the trends are presented in Table S1.

**Figure 2:**
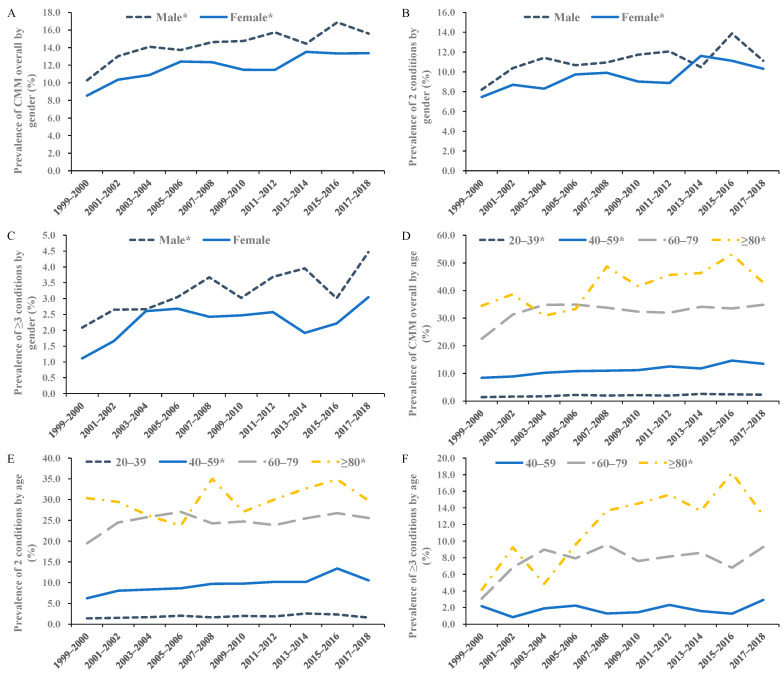
Trends in the age-adjusted prevalence of cardiometabolic multimorbidity by gender and age among U.S. adults, 1999–2018. Notes: The averaged trends were estimated with AAPC derived from joinpoint regression models. Panel (**A**): For CMM overall by gender; Panel (**B**): For concurrent two cardiometabolic diseases by gender; Panel (**C**): For ≥three concurrent cardiometabolic diseases by gender; Panel (**D**): For CMM overall by age; Panel (**E**): For two concurrent cardiometabolic diseases by age; Panel (**F**): For ≥three concurrent cardiometabolic diseases by age. The prevalence of 20–39 years subgroup for ≥three conditions during 1999–2018 was too low to be estimated, which hindered the estimation of AAPC with joinpoint regression models, so the related trend is not shown in the figure. CMM, cardiometabolic multimorbidity; AAPC, averaged two-year cycle percentage change. * *p* for trend <0.05.

**Figure 3:**
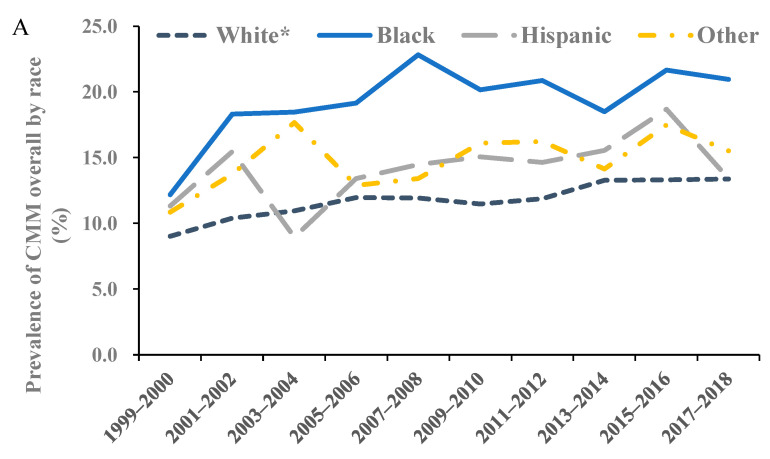

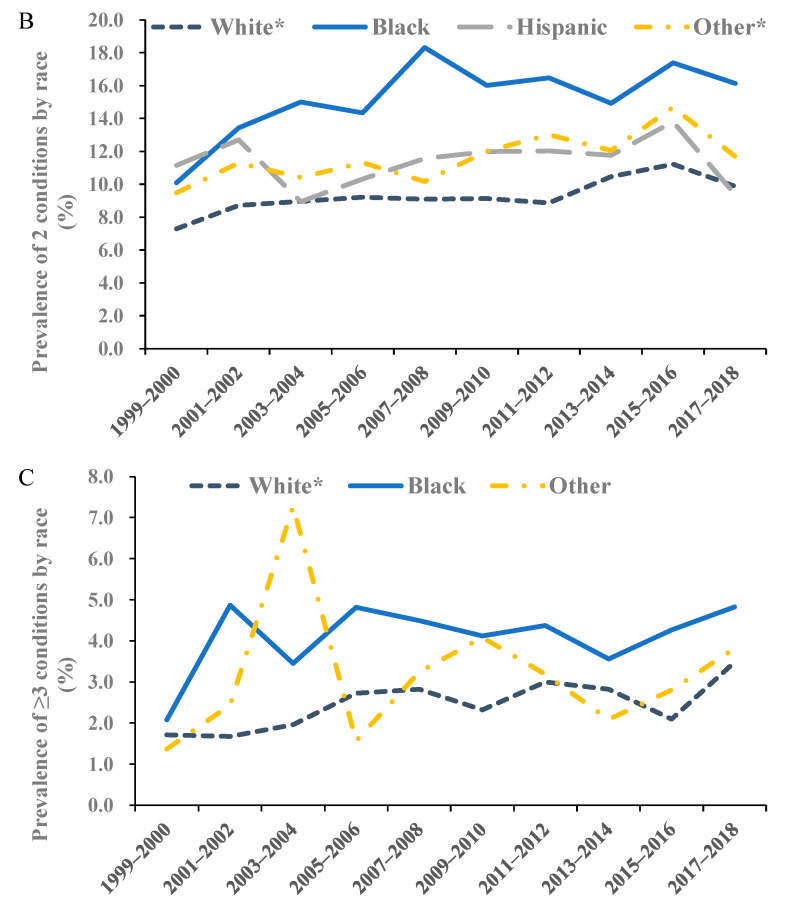
Trends in the age-adjusted prevalence of cardiometabolic multimorbidity by race/ethnicity among U.S. adults, 1999–2018. Notes: The averaged trends were estimated with AAPC derived from joinpoint regression models. Panel (**A**): For CMM overall by race/ethnicity; Panel (**B**): For two concurrent cardiometabolic diseases by race/ethnicity; Panel (**C**): For ≥three concurrent cardiometabolic diseases by race/ethnicity. The prevalence of Hispanic subgroup for ≥three conditions during 1999–2018 was too low to be estimated, which hindered the estimation of AAPC with joinpoint regression models, so the related trend is not shown in the figure. CMM, cardiometabolic multimorbidity; AAPC, averaged two-year cycle percentage change. * *p* for trend <0.05.

When stratified by gender, the prevalence of cardiometabolic multimorbidity overall in both genders increased, while that of two diseases increased significantly for females (AAPC: 3.9, 95% CI: 1.5 to 6.4), and ≥three conditions increased for males (AAPC: 5.7, 95% CI: 2.2 to 9.3) (Figure 2A–C). In most age groups except for 60–79 years, the prevalence of cardiometabolic multimorbidity overall increased steadily (Figure 2D). For two conditions (Figure 2E), the prevalence in people aged 40–59 and ≥80 years increased significantly (AAPC: 6.2 (95% CI: 3.1 to 9.5) for 40–59 years; 2.6 (95% CI: 0.1 to 5.1) for ≥80 years). Notably, the prevalence of ≥three conditions (Figure 2F) in the population aged 80 years and older nearly tripled from 4.1% in 1999–2000 to 13.1% in 2017–2008, with an AAPC of 9.4 (95% CI: 1.6 to 17.9). The trend in the non-Hispanic White population was increasing significantly (AAPC: 3.7, 95% CI: 2.4 to 5.1 for cardiometabolic multimorbidity overall), while the increasing trends in other races did not reach statistical significance (Figure 3), except for the prevalence of two cardiometabolic diseases among people of “other” race/ethnicity. However, the absolute value of the prevalence of cardiometabolic multimorbidity among non-Hispanic Black people remained the highest during 1999–2018. The values of the prevalence and AAPC for the trends in subgroups by gender, age, and race/ethnicity are presented in Tables S2–S4. In addition, according to the results derived from the Joinpoint Regression Program, adding one joinpoint was significant to fit trend models for certain patterns and subgroups (i.e., two segments), and the detailed information, segmental APC, and *p* value of these models are available in Table S5.

## 4. Discussion

### 4.1. Main Findings

Based on the representative population from the NHANES, this study analyzed the trends in the prevalence of cardiometabolic multimorbidity in U.S. adults from 1999 to 2018. The latest prevalence of cardiometabolic multimorbidity was high among U.S. adults, especially in the male, older, and non-Hispanic Black population. The prevalence of specific patterns of cardiometabolic multimorbidity varied widely, with nearly 50% of patients having hypertension and diabetes. From 1999 to 2018, the prevalence of cardiometabolic multimorbidity overall in U.S. adults increased from 9.4% to 14.4%, and the increasing trend was significant for both genders, all age groups except for “60–79 years” and non-Hispanic White population. For three common patterns, the trend was increasing for hypertension and diabetes and hypertension, diabetes, and CHD, while it was decreasing for hypertension and CHD.

### 4.2. Comparison with Previous Researches

According to our estimates, the prevalence of cardiometabolic multimorbidity in the entire U.S. population was as high as 14.4% during 2017–2018. Combined with previous research conducted in other areas, such as China [3,14], Canada [12], and South Asia [13], we observed a wide variety in the reported prevalence of this comorbidity. For instance, in a longitudinal study of 1,038,704 Chinese adults, Zhang et.al. reported that 5.9% of participants suffered from cardiometabolic multimorbidity in 2016 [14], while a recent study reported the prevalence was 24.5% among middle-aged and older Chinese individuals [3]. It was estimated that 3.5% (467,749 individuals) of Canadians were affected by cardiometabolic multimorbidity in 2016 [12], while in rural South Asia, the relevant prevalence in hypertensive patients was ~25.4% [13]. A main source of the discrepancy was the different definitions of cardiometabolic multimorbidity used in each study. Although cardiometabolic diseases include hypertension, diabetes, CHD, heart failure, chronic kidney disease, dyslipidemia, etc., most research involved three or four common diseases with crucial clinical outcomes because of data availability or study designing [6], leading to the diversity in the comorbidity definition and prevalence. The population characteristics also contributed to the diversity of prevalence. Some studies were conducted based on older people [3,12] or patients with hypertension [13] who were prone to have cardiometabolic diseases, hindering the estimation of cardiometabolic multimorbidity prevalence at an area level. In this study, based on the nationally representative NHANES survey data, we found that the prevalence in U.S. adults was 14.4%, and 30%~40% of older people (≥60 years) suffered from comorbidity, which was substantially higher compared with that of other areas, even considering the differences in study settings and population features.

The prevalence of cardiometabolic multimorbidity in U.S. adults increased steadily from 9.4% in 1999–2000 to 14.4% in 2017–2018, with a relative 3.6% change per two-year cycle. The increasing trend was also observed in China, as the prevalence doubled over five years since 2010 [14]. When analyzed individually, the prevalence of most cardiometabolic diseases involved in the current study increased during the past decade, except for heart diseases [22,23]. With the development of medical technologies, the coexistence of several chronic diseases, i.e., multimorbidity, has been common, with the tendency of global aging exacerbating the situation [24]. Considering that cardiometabolic diseases share common and overlapped risk factors, etiology, and metabolic disorders, a rapid increase in the prevalence of cardiometabolic multimorbidity is expected [7], and our study provided intuitive evidence for this heavy disease burden in the U.S. Furthermore, we estimated the trends in the prevalence of three common patterns of cardiometabolic multimorbidity and observed that it was increasing for hypertension and diabetes (4.3% in 1999–2000; 7.5% in 2017–2018) and hypertension, diabetes, and CHD (0.8% in 1999–2000; 2.2% in 2017–2018), while it was descending for hypertension and CHD (2.0% in 1999–2000; 1.8% in 2017–2018). The opposite trend between the latter two patterns hinted at a substantial transition from two concurrent diseases to three diseases during the past two decades, which should be more concerning since the additive effects of cardiometabolic multimorbidity have been observed on all-cause mortality, mental disorders, and life quality [3,6,25].

Considering the high and increasing prevalence of cardiometabolic multimorbidity, cost-effective strategies should be implemented to prevent its incidence and improve prognosis. Existing studies suggested that clinical factors, lifestyles, and socioeconomic levels exhibited distinct effects on the progression and prognosis of cardiometabolic multimorbidity [26,27] and that it is improper to target risk or protective factors of single cardiometabolic diseases directly. However, the majority of previous studies focused on the primary prevention of cardiometabolic multimorbidity, and only a few studies explored the secondary prevention of cardiometabolic multimorbidity. Moreover, results about secondary prevention varied widely among studies. For instance, some studies found that regular physical activity and non-smoking decreased the risk of mortality in patients with cardiometabolic multimorbidity, while others found that the associations between regular physical activity or non-smoking and mortality were not statistically significant [26,28,29]. Therefore, more studies about the secondary prevention of cardiometabolic multimorbidity are needed in future research. In addition to focusing on the strategies specified for this comorbidity, the appropriate control of single conditions that have occurred is also necessary. For patients who already have diabetes or hypertension, effective interventions to control glycemic and blood pressure levels are recommended by guidelines to prevent new-onset cardiovascular diseases [30,31]. However, during 2015–2018, ~35% of adults with diagnosed diabetes in the U.S. did not achieve HbA1c control goals, and more than 50% of diabetic patients did not have well-controlled blood pressure levels [32]. Among hypertensive patients, the rate of blood pressure control was 40%~50% in 2014 [33]. For both policy makers and individuals, the unsatisfactory control rate of glycemic and blood pressure levels highlights potential opportunities to take steps to postpone the transition from single disease to cardiometabolic multimorbidity and, therefore, alleviate the heavy burden of cardiometabolic diseases.

In stratified analyses by gender, age, and race/ethnicity, we identified subgroups that need more attention in the prevention and intervention of cardiometabolic multimorbidity. In 2017–2018, the prevalence of cardiometabolic multimorbidity in men was slightly higher than that in women. From 1999–2000 to 2017–2018, the prevalence of three concurrent cardiometabolic diseases in the age group of ≥80 years nearly tripled, with a relative 9.4% increase per two-year cycle. Even though the increasing trend in the subgroup of 60–79 years did not reach significance, 34.8% of people aged 60–79 and 42.9% of people aged 80 years and older suffer from this comorbidity in 2017–2018. Among race/ethnicity subgroups, the overall prevalence among non-Hispanic Black people remained the highest during the past twenty years, while among non-Hispanic White people it increased significantly. The disparity in the prevalence and trends among subgroups indicated that targeted policies, education, and interventions are necessary to cope with this distressing issue.

### 4.3. Strengths and Limitations

The strengths and limitations of this study deserve comments. Based on the nationally representative data from 10 NHANES two-year cycles, our results could be generalized to the entire U.S. adults and provided evidence for the increasing trend of cardiometabolic multimorbidity during such a long period. The stratified analyses according to gender, age, and race/ethnicity also assisted the identification of subgroups that were at a higher risk of this comorbidity and needed more attention. Nevertheless, self-reported medical history, medication use, and single-occasion laboratory measurements were relied on to assess the status of cardiometabolic diseases, which could contribute to the misclassification of diseases. With samples from cross-sectional surveys, we were unable to trace the transition of the healthy or single disease to multiple diseases, but the serial data enabled us to investigate the trends and prevalence of cardiometabolic multimorbidity across the whole U.S. during the past two decades. Another limitation was that the sample size limited detailed stratification analyses for trends and prevalence of specific patterns. We reported the prevalence and trends of cardiometabolic multimorbidity overall and for two or ≥three conditions by gender, age, and race/ethnicity, but the results should be interpreted carefully, especially for subgroups in which the case number was <20 (tagged with “unreliable” in our results). Rather than emphasizing the prevalence and trends of each subgroup, it is more advisable to consider the overall prevalence and trends and to pay attention to subgroups with higher prevalence or more rapidly increasing trends. Additionally, there has been no consensus definition of cardiometabolic multimorbidity. To make our results comparable with studies conducted in other regions, we determined cardiometabolic diseases involved in this research based on most published studies in this comorbidity, while other cardiometabolic conditions, such as peripheral artery diseases and dyslipidemia, were not considered in our analyses. Thus, the results should be carefully interpreted and compared with other studies, accounting for the potential differences in the definition of comorbidity.

## 5. Conclusions

Based on the nationally representative NHANES data, we observed that from 1999 to 2018, the prevalence of cardiometabolic multimorbidity increased significantly. In 2017–2018, the latest prevalence among U.S. adults was high, especially in the male, older, and non-Hispanic Black population. The prevalence varied widely among specific cardiometabolic multimorbidity patterns, with nearly 50% of patients having hypertension and diabetes. During the past two decades, the prevalence increased for patterns of hypertension and diabetes and hypertension, diabetes, and CHD, while decreasing for hypertension and CHD. Our findings provided evidence that cardiometabolic multimorbidity has risen as an austere issue of public health in the United States.

## Figures and Tables

**Table 1:** The prevalence of cardiometabolic multimorbidity among adults in the U.S., 2017–2018.

Characteristics	Overall		Two Diseases		≥Three Diseases	
	*n* = 523		*n* = 385		*n* = 138	
	Prevalence, % (95% CI) ^1^	Estimated Counts, Million	Prevalence, % (95% CI) ^1^	Estimated Counts, Million	Prevalence, % (95% CI) ^1^	Estimated Counts, Million
Total	14.4 (12.6 to 16.1)	34.27	10.7 (9.5 to 11.9)	25.47	3.7 (2.8 to 4.6)	8.80
Age, year						
20–39 ^2^	2.3 (1.0 to 3.6)	5.49	1.6 (1.0 to 2.2)	3.81	\	\
40–59	13.5 (9.9 to 17.1)	32.16	10.6 (8.2 to 12.9)	25.19	2.9 (1.1 to 4.7)	6.97
60–79	34.8 (29.0 to 40.6)	83.14	25.5 (20.2 to 30.8)	60.94	9.3 (6.5 to 12.1)	22.20
≥80	42.9 (34.5 to 51.2)	102.33	29.8 (23.9 to 35.6)	71.10	13.1 (8.1 to 18.1)	31.23
Gender						
Male	15.6 (13.3 to 17.8)	37.23	11.1 (9.4 to 12.9)	26.56	4.5 (3.1 to 5.8)	10.68
Female	13.4 (11.0 to 15.7)	31.91	10.3 (8.3 to 12.3)	24.64	3.0 (2.0 to 4.1)	7.27
Race/ethnicity						
White	13.4 (11.1 to 15.6)	31.90	9.9 (8.2 to 11.6)	23.60	3.5 (2.6 to 4.4)	8.31
Black	21.0 (17.4 to 24.5)	50.04	16.1 (13.6 to 18.7)	38.51	4.8 (3.6 to 6.1)	11.54
Hispanic	13.4 (9.5 to 17.4)	32.01	9.3 (7.2 to 11.5)	22.32	4.1 (1.3 to 6.8)	9.69
Other ^3^	15.5 (12.5 to 18.6)	37.02	11.7 (9.2 to 14.2)	27.90	3.8 (2.0 to 5.7)	9.12

^1^ Age-standardized prevalence. ^2^ Labeled when the case number of this subgroup is <20 and caution is needed to interpretation. The results are not reported if the lower value of the corresponding prevalence was <0 or the upper value was = 0. ^3^ Other race/ethnicity included Mexican American and Other race (including multi-race) people defined in NHANES. NHANES, National Health and Nutrition Examination Survey.

## Data Availability

This study utilizes publicly available data, which can be accessed via https://wwwn.cdc.gov/nchs/nhanes/, accessed on 18 January 2022.

## Supplementary Materials

The following supporting information can be downloaded at https://www.mdpi.com/article/10.3390/ijerph19084726/s1, Table S1: Trends in the prevalence of cardiometabolic multimorbidity and common patterns among U.S. adults, 1999–2018; Table S2: Trends in the prevalence of cardiometabolic multimorbidity by gender among U.S. adults, 1999–2018; Table S3: Trends in the prevalence of cardiometabolic multimorbidity by age among U.S. adults, 1999–2018; Table S4: Trends in the prevalence of cardiometabolic multimorbidity by race/ethnicity among U.S. adults, 1999–2018; Table S5: Trends of cardiometabolic multimorbidity among certain populations with one joinpoint, 1999–2018.
