# Supplementary material for: Trends in the Prevalence of Cardiometabolic Multimorbidity in the United States, 1999–2018
Source: Int J Environ Res Public Health. 2022 Apr 14;19(8):4726. doi: 10.3390/ijerph19084726 (PMC9027860; doi:10.3390/ijerph19084726)
Supplement: Supplementary file 1 [file ijerph-19-04726-s001.zip › ijerph-1641067-supplementary.pdf]

Table S1. Trends in the prevalence of cardiometabolic multimorbidity and common patterns among U.S. adults, 1999-2018

Characteristics	Prevalence of cardiometabolic multimorbidity, % (95% CI) [1]										AAPC (95% CI) [2]	*P*
	1999-2000	2001-2002	2003-2004	2005-2006	2007-2008	2009-2010	2011-2012	2013-2014	2015-2016	2017-2018		
Total number	1852	2199	1974	1979	2355	2619	2313	2389	2268	2291		
Overall	9.4 (7.8 to 11.0)	11.5 (9.9 to 13.2)	12.4 (10.1 to 14.6)	13.0 (11.0 to 14.9)	13.4 (11.5 to 15.3)	12.9 (11.7 to 14.1)	13.4 (11.5 to 15.4)	13.9 (12.3 to 15.5)	15.0 (13.1 to 16.9)	14.4 (12.6 to 16.1)	**3.6 (2.1 to 5.3)**	**0.001**
2 diseases	7.9 (6.5 to 9.2)	9.5 (7.9 to 11.1)	9.8 (8.0 to 11.6)	10.1 (8.5 to 11.8)	10.4 (9.1 to 11.7)	10.2 (9.2 to 11.3)	10.3 (8.7 to 11.9)	11.1 (9.7 to 12.4)	12.4 (10.6 to 14.3)	10.7 (9.5 to 11.9)	**2.9 (1.1 to 4.7)**	**0.006**
≥3 diseases	1.6 (1.1 to 2.0)	2.1 (1.5 to 2.7)	2.6 (1.7 to 3.5)	2.8 (2.0 to 3.7)	3.0 (2.0 to 4.0)	2.7 (2.2 to 3.1)	3.1 (2.4 to 3.9)	2.8 (1.8 to 3.8)	2.6 (1.9 to 3.2)	3.7 (2.8 to 4.6)	**6.1 (1.9 to 10.4)**	**0.009**
Hypertension and diabetes	4.3 (2.9 to 5.8)	6.0 (4.4 to 7.5)	5.4 (4.2 to 6.7)	6.1 (5.0 to 7.3)	6.8 (6.2 to 7.4)	7.2 (6.2 to 8.2)	6.9 (5.6 to 8.3)	7.6 (6.5 to 8.6)	8.8 (7.2 to 10.4)	7.5 (6.5 to 8.6)	**4.8 (2.4 to 7.2)**	**0.002**
Hypertension, diabetes, and CHD	0.8 (0.3 to 1.3)	1.0 (0.7 to 1.3)	1.3 (0.6 to 1.9)	1.1 (0.6 to 1.6)	1.5 (1.0 to 2.1)	1.5 (1.2 to 1.7)	1.8 (1.3 to 2.4)	1.6 (1.0 to 2.1)	1.3 (0.7 to 1.8)	2.2 (1.4 to 3.0)	**8.7 (3.8 to 13.7)**	**0.003**
Hypertension and CHD	2.0 (1.3 to 2.7)	2.4 (1.7 to 3.0)	2.6 (1.9 to 3.2)	2.5 (1.8 to 3.2)	2.2 (1.6 to 2.8)	1.9 (1.4 to 2.4)	1.9 (1.4 to 2.4)	1.7 (1.4 to 2.0)	2.3 (1.4 to 3.1)	1.8 (1.0 to 2.6)	**-4.1 (-7.3 to -0.8)**	**0.022**

[1] Age-standardized prevalence.

[2] AAPC means averaged 2-year cycle percentage change.

Boldface indicates statistical significance (*P*<0.05).

CHD, coronary heart disease.

Table S2. Trends in the prevalence of cardiometabolic multimorbidity by gender among U.S. adults, 1999-2018

Prevalence, % (95% CI) [1]	Gender	1999-2000	2001-2002	2003-2004	2005-2006	2007-2008	2009-2010	2011-2012	2013-2014	2015-2016	2017-2018	AAPC (95% CI) [2]	*P*
Overall	Male	10.3 (7.3 to 13.3)	13.0 (10.6 to 15.4)	14.1 (11.4 to 16.8)	13.7 (11.5 to 15.9)	14.6 (12.6 to 16.7)	14.8 (12.9 to 16.6)	15.7 (13.4 to 18.1)	14.4 (12.5 to 16.3)	16.9 (14.5 to 19.3)	15.6 (13.3 to 17.8)	**2.9 (1.1 to 4.8)**	**0.006**
	Female	8.6 (7.0 to 10.1)	10.4 (8.5 to 12.2)	10.9 (8.6 to 13.2)	12.4 (9.9 to 15.0)	12.3 (9.6 to 15.1)	11.5 (9.6 to 13.3)	11.5 (9.4 to 13.5)	13.5 (11.6 to 15.5)	13.3 (10.8 to 15.9)	13.4 (11 to 15.7)	**4.2 (2.2 to 6.3)**	**0.001**
2 diseases	Male	8.2 (6.3 to 10.1)	10.4 (8.2 to 12.6)	11.4 (9.0 to 13.8)	10.7 (9.2 to 12.2)	11.0 (9.5 to 12.4)	11.7 (9.9 to 13.6)	12.1 (9.7 to 14.4)	10.5 (9.0 to 12.0)	13.9 (11.6 to 16.2)	11.1 (9.4 to 12.9)	2.4 (-0.4 to 5.2)	0.083
	Female	7.4 (5.8 to 9.1)	8.7 (6.9 to 10.5)	8.3 (6.7 to 9.8)	9.7 (7.5 to 12.0)	9.9 (7.8 to 12.0)	9.0 (7.5 to 10.6)	8.9 (7.1 to 10.7)	11.6 (9.9 to 13.3)	11.1 (8.7 to 13.6)	10.3 (8.3 to 12.3)	**3.9 (1.5 to 6.4)**	**0.005**
≥3 diseases	Male	2.1 (0.7 to 3.5)	2.7 (1.8 to 3.5)	2.7 (1.9 to 3.5)	3.1 (2.0 to 4.1)	3.7 (2.1 to 5.2)	3.0 (2.3 to 3.8)	3.7 (2.4 to 5.0)	4.0 (2.4 to 5.5)	3.0 (2.0 to 4.0)	4.5 (3.1 to 5.8)	**5.7 (2.2 to 9.3)**	**0.005**
	Female	1.1 (0.3 to 1.9)	1.7 (1.0 to 2.3)	2.6 (1.4 to 3.8)	2.7 (1.5 to 3.9)	2.4 (1.3 to 3.5)	2.5 (1.8 to 3.1)	2.6 (1.7 to 3.4)	1.9 (1.0 to 2.9)	2.2 (1.4 to 3.0)	3.0 (2.0 to 4.1)	4.6 (-1.1 to 10.5)	0.100

[1] Age-standardized prevalence.

[2] AAPC means averaged 2-year cycle percentage change.

Boldface indicates statistical significance (*P*<0.05).

Table S3. Trends in the prevalence of cardiometabolic multimorbidity by age among U.S. adults, 1999-2018

Prevalence, % (95% CI) [1]	Age, year	1999-2000	2001-2002	2003-2004	2005-2006	2007-2008	2009-2010	2011-2012	2013-2014	2015-2016	2017-2018	AAPC (95% CI) [3]	*P*
Overall	20-39 [2]	1.4 (0.6 to 2.2)	1.7 (0.6 to 2.8)	1.7 (0.5 to 2.9)	2.2 (1.2 to 3.3)	2.0 (1.2 to 2.7)	2.2 (0.8 to 3.5)	2.0 (1.0 to 3.0)	2.6 (0.8 to 4.3)	2.4 (0.9 to 4.0)	2.3 (1.0 to 3.6)	**5.2 (2.0 to 8.5)**	**0.005**
	40-59	8.4 (5.6 to 11.2)	8.9 (5.9 to 11.8)	10.2 (7.2 to 13.2)	10.9 (7.6 to 14.1)	11.0 (8.6 to 13.4)	11.2 (9.7 to 12.7)	12.5 (9.5 to 15.6)	11.8 (9 to 14.6)	14.7 (12.4 to 16.9)	13.5 (9.9 to 17.1)	**6.0 (4.1 to 7.9)**	**<0.001**
	60-79	22.6 (16.4 to 28.8)	31.3 (27.1 to 35.5)	34.8 (28.3 to 41.4)	35.0 (28.7 to 41.2)	33.8 (28.6 to 39.1)	32.3 (27.4 to 37.3)	32.0 (27.2 to 36.8)	34.1 (28.8 to 39.3)	33.5 (29.4 to 37.6)	34.8 (29.0 to 40.6)	1.3 (-0.8 to 3.4)	0.185
	≥80	34.5 (24.3 to 44.6)	38.7 (31.6 to 45.8)	30.9 (25.5 to 36.3)	33.3 (23.2 to 43.5)	48.7 (39.3 to 58.1)	41.6 (31.8 to 51.3)	45.7 (35.0 to 56.3)	46.3 (37.3 to 55.4)	53 (44.7 to 61.4)	42.9 (34.5 to 51.2)	**4.5 (1.0 to 8.1)**	**0.018**
2 diseases	20-39 [2]	1.4 (0.6 to 2.2)	1.5 (0.5 to 2.5)	1.7 (0.5 to 2.9)	2.0 (1.1 to 2.9)	1.6 (0.9 to 2.3)	2.0 (0.7 to 3.2)	1.9 (0.9 to 2.9)	2.6 (0.8 to 4.3)	2.3 (0.8 to 3.8)	1.6 (1.0 to 2.2)	1.9 (-2.5 to 6.6)	0.351
	40-59	6.2 (3.7 to 8.7)	8.0 (5.3 to 10.8)	8.3 (5.4 to 11.3)	8.6 (6.4 to 10.9)	9.7 (7.8 to 11.6)	9.8 (8.4 to 11.1)	10.2 (6.8 to 13.6)	10.2 (7.9 to 12.5)	13.4 (11.2 to 15.6)	10.6 (8.2 to 12.9)	**6.2 (3.1 to 9.5)**	**0.002**
	60-79	19.5 (13.5 to 25.5)	24.5 (19.1 to 29.8)	25.8 (21.1 to 30.6)	27.0 (21.6 to 32.4)	24.3 (20.2 to 28.3)	24.7 (19.5 to 29.9)	23.8 (19.5 to 28.2)	25.5 (22.5 to 28.5)	26.7 (22.8 to 30.6)	25.5 (20.2 to 30.8)	1.0 (-0.7 to 2.8)	0.215
	≥80	30.3 (19.0 to 41.7)	29.4 (23.0 to 35.9)	26.0 (23.1 to 28.9)	23.7 (14.7 to 32.8)	35.0 (24.8 to 45.3)	27.1 (18.6 to 35.5)	30.1 (19.0 to 41.2)	32.7 (28.0 to 37.5)	34.8 (27.8 to 41.8)	29.8 (23.9 to 35.6)	**2.6 (0.1 to 5.1)**	**0.040**

≥3 diseases	20-39 [2]	\	\	\	\	0.4 (0.0 to 0.7)	\	\	\	\	\	---	---
	40-59 [2]	2.2 (1.2 to 3.1)	0.8 (0.2 to 1.4)	1.9 (0.8 to 3.0)	2.2 (0.7 to 3.8)	1.3 (0.3 to 2.3)	1.4 (0.5 to 2.3)	2.3 (0.4 to 4.3)	1.6 (0.1 to 3.0)	1.2 (0.6 to 1.9)	2.9 (1.1 to 4.7)	0.4 (-7.9 to 9.5)	0.913
	60-79	3.1 (1.2 to 5)	6.8 (4.2 to 9.4)	9.0 (5.8 to 12.2)	7.9 (5.3 to 10.6)	9.6 (5.6 to 13.5)	7.6 (6.0 to 9.2)	8.2 (4.5 to 11.8)	8.6 (4.8 to 12.4)	6.8 (4.6 to 9.0)	9.3 (6.5 to 12.1)	2.9 (-3.0 to 9.1)	0.295
	≥80 [2]	4.1 (1.2 to 7.1)	9.3 (5.2 to 13.3)	4.9 (1.0 to 8.7)	9.6 (5.0 to 14.2)	13.7 (9.6 to 17.7)	14.5 (8.5 to 20.5)	15.6 (8.3 to 22.9)	13.6 (6.9 to 20.3)	18.2 (10.4 to 26.1)	13.1 (8.1 to 18.1)	**9.4 (1.6 to 17.9)**	**0.024**

[1] Age-standardized prevalence.

[2] Labelled when the case number of this subgroup <20, and caution is needed to interpretation. The results are not reported if the lower value of corresponding prevalence was <0 or the upper value was =0.

[3] AAPC means averaged 2-year cycle percentage change.

Boldface indicates statistical significance ($P<0.05$).

--- The prevalence of this subgroup during 1999-2018 was too low to be estimated, which hindered the estimation of AAPC with Joinpoint regression models.

Table S4. Trends in the prevalence of cardiometabolic multimorbidity by race/ethnicity among U.S. adults, 1999-2018

Prevalence, % (95% CI) [1]	Race	1999-2000	2001-2002	2003-2004	2005-2006	2007-2008	2009-2010	2011-2012	2013-2014	2015-2016	2017-2018	AAPC (95% CI) [3]	*P*
Overall	White	9.0 (7.1 to 11.0)	10.4 (8.6 to 12.2)	10.9 (8.6 to 13.3)	11.9 (9.5 to 14.3)	11.9 (9.5 to 14.4)	11.5 (9.9 to 13)	11.9 (9.6 to 14.1)	13.3 (11 to 15.6)	13.3 (10.5 to 16.1)	13.4 (11.1 to 15.6)	**3.7 (2.4 to 5.1)**	**<0.001**
	Black	12.2 (8.2 to 16.1)	18.3 (14.4 to 22.2)	18.5 (15.7 to 21.2)	19.2 (15.1 to 23.2)	22.8 (18.4 to 27.3)	20.1 (17.5 to 22.8)	20.8 (17.5 to 24.2)	18.5 (15.9 to 21.1)	21.7 (18.7 to 24.6)	21.0 (17.4 to 24.5)	2.3 (-0.5 to 5.2)	0.093
	Hispanic [2]	11.3 (7.1 to 15.5)	15.4 (10.1 to 20.8)	\	13.4 (3.4 to 23.5)	14.5 (10.2 to 18.8)	15.0 (8.9 to 21.1)	14.6 (9.3 to 19.9)	15.5 (9.5 to 21.6)	18.7 (15.1 to 22.3)	13.4 (9.5 to 17.4)	3.0 (-0.7 to 6.9)	0.098
	Other [4]	10.9 (7.4 to 14.3)	13.7 (9.9 to 17.6)	17.7 (12.1 to 23.3)	12.9 (7.8 to 18.0)	13.4 (8.5 to 18.3)	16.1 (12.0 to 20.2)	16.2 (12.5 to 19.9)	14.1 (11.3 to 17.0)	17.5 (14.6 to 20.3)	15.5 (12.5 to 18.6)	2.7 (-0.4 to 5.8)	0.079
2 diseases	White	7.3 (5.7 to 8.9)	8.7 (7 to 10.4)	9.0 (7.0 to 11.0)	9.2 (7.2 to 11.2)	9.1 (7.2 to 11.0)	9.1 (7.8 to 10.5)	8.9 (7.1 to 10.6)	10.5 (8.6 to 12.3)	11.2 (8.6 to 13.8)	9.9 (8.2 to 11.6)	**2.9 (1.1 to 4.7)**	**0.005**
	Black	10.1 (6.7 to 13.5)	13.4 (10.4 to 16.5)	15.0 (12.0 to 18.1)	14.3 (10.7 to 17.9)	18.3 (14.9 to 21.8)	16.0 (13.3 to 18.7)	16.5 (12.8 to 20.1)	14.9 (11.3 to 18.6)	17.4 (15.1 to 19.7)	16.1 (13.6 to 18.7)	2.7 (-0.1 to 5.6)	0.060
	Hispanic [2]	11.2 (7.1 to 15.2)	12.7 (7.0 to 18.4)	\	10.3 (2.3 to 18.3)	11.6 (8.7 to 14.5)	12.0 (7.3 to 16.7)	12.0 (7.1 to 16.9)	11.8 (6.3 to 17.2)	13.8 (10.7 to 16.9)	9.3 (7.2 to 11.5)	-0.6 (-4.3 to 3.2)	0.714

	Other [4]	9.5 (6.1 to 12.9)	11.3 (7.5 to 15.1)	10.4 (6.7 to 14.1)	11.3 (7.4 to 15.3)	10.2 (5.8 to 14.6)	12.0 (8.3 to 15.7)	13.0 (9.3 to 16.8)	12.1 (9.3 to 14.8)	14.7 (11.9 to 17.5)	11.7 (9.2 to 14.2)	**3.0 (0.3 to 5.7)**	**0.031**
≥3 diseases	White	1.7 (1.2 to 2.3)	1.7 (0.9 to 2.4)	2.0 (1.1 to 2.8)	2.7 (1.6 to 3.8)	2.8 (1.7 to 4.0)	2.3 (1.9 to 2.8)	3.0 (2.1 to 3.9)	2.8 (1.5 to 4.1)	2.1 (1.3 to 2.9)	3.5 (2.6 to 4.4)	**6.8 (2.5 to 11.3)**	**0.006**
	Black [2]	2.1 (0.9 to 3.2)	4.9 (1.6 to 8.1)	3.5 (1.5 to 5.4)	4.8 (2.6 to 7.0)	4.5 (2.2 to 6.8)	4.1 (2.2 to 6.0)	4.4 (2.6 to 6.2)	3.6 (1.6 to 5.5)	4.3 (2.6 to 5.9)	4.8 (3.6 to 6.1)	4.1 (-0.8 to 9.3)	0.091
	Hispanic [2]	\	2.7 (1.2 to 4.3)	\	\	2.9 (1.1 to 4.8)	\	2.6 (1.3 to 4.0)	3.8 (0.6 to 6.9)	4.9 (2.8 to 7.0)	4.1 (1.3 to 6.8)	---	---
	Other [2, 4]	1.4 (0.6 to 2.2)	2.4 (0.6 to 4.3)	7.3 (1.9 to 12.7)	\	3.2 (1.9 to 4.6)	4.1 (2.1 to 6.1)	3.2 (2.4 to 4.0)	2.1 (1.0 to 3.2)	2.8 (0.9 to 4.7)	3.8 (2.0 to 5.7)	4.5 (-6.4 to 16.6)	0.387

[1] Age-standardized prevalence.

[2] Labelled when the case number of this subgroup <20, and caution is needed to interpretation. The results are not reported if the lower value of corresponding prevalence was <0 or the upper value was =0.

[3] AAPC means averaged 2-year cycle percentage change.

[4] Other race/ethnicity included Mexican American and Other race (including multi-race) people defined in NHANES.

Boldface indicates statistical significance (*P*<0.05).

--- The prevalence of this subgroup during 1999-2018 was too low to be estimated, which hindered the estimation of AAPC with Joinpoint regression models.

NHANES, National Health and Nutrition Examination Survey.

Table S5. Trends of cardiometabolic multimorbidity among certain population with 1 joinpoint, 1999-2018

Characteristics	Subgroup	Segment	Lower Endpoint (year)	Upper Endpoint (year)	APC (95% CI) [1]	*P*
Overall cardiometabolic multimorbidity	Total population	1	1999-2000	2003-2004	13.4 (-2.7 to 32.2)	0.089
		2	2003-2004	2017-2018	**2.2 (0.5 to 4)**	**0.023**
2 cardiometabolic diseases	Female	1	1999-2000	2003-2004	49.3 (-32.8 to 231.7)	0.253
		2	2003-2004	2017-2018	0.3 (-6.8 to 7.9)	0.926
Overall cardiometabolic multimorbidity	60-79 years	1	1999-2000	2003-2004	16.6 (-3 to 40.2)	0.085
		2	2003-2004	2017-2018	-0.5 (-2.3 to 1.3)	0.473

[1] APC means 2-year cycle percentage change.

Boldface indicates statistical significance (*P*<0.05).
